# Antioxidant Activity of Selected Phenolic Acids–Ferric Reducing Antioxidant Power Assay and QSAR Analysis of the Structural Features

**DOI:** 10.3390/molecules25133088

**Published:** 2020-07-07

**Authors:** Maciej Spiegel, Karina Kapusta, Wojciech Kołodziejczyk, Julia Saloni, Beata Żbikowska, Glake A. Hill, Zbigniew Sroka

**Affiliations:** 1 Department of Pharmacognosy, Wroclaw Medical University, Borowska 211, 50-556 Wroclaw, Poland; maciej.spiegel@student.umed.wroc.pl (M.S.); beata.zbikowska@umed.wroc.pl (B.Ż.); z.g.sroka@gmail.com (Z.S.); 2 Interdisciplinary Center for Nanotoxicity, Department of Chemistry, Physics and Atmospheric Sciences, Jackson State University, 1400 J. R. Lynch str., Jackson, MS 39217, USA; dziecial@icnanotox.org (W.K.); julia.m.saloni@jsums.edu (J.S.); glakeh@icnanotox.org (G.A.H.)

**Keywords:** phenolic acids, polyphenols, antioxidants, ferric reducing antioxidant power (FRAP) assay, density functional theory (DFT), quantitative structure–activity relationship (QSAR), multiple linear regression (MLR)

## Abstract

Phenolic acids are naturally occurring compounds that are known for their antioxidant and antiradical activity. We present experimental and theoretical studies on the antioxidant potential of the set of 22 phenolic acids with different models of hydroxylation and methoxylation of aromatic rings. Ferric reducing antioxidant power assay was used to evaluate this property. 2,3-dihydroxybenzoic acid was found to be the strongest antioxidant, while mono hydroxylated and methoxylated structures had the lowest activities. A comprehensive structure–activity investigation with density functional theory methods elucidated the influence of compounds topology, resonance stabilization, and intramolecular hydrogen bonding on the exhibited activity. The key factor was found to be a presence of two or more hydroxyl groups being located in *ortho* or *para* position to each other. Finally, the quantitative structure–activity relationship approach was used to build a multiple linear regression model describing the dependence of antioxidant activity on structure of compounds, using features exclusively related to their topology. Coefficients of determination for training set and for the test set equaled 0.9918 and 0.9993 respectively, and Q2 value for leave-one-out was 0.9716. In addition, the presented model was used to predict activities of phenolic acids that haven’t been tested here experimentally.

## 1. Introduction

Nowadays, the popularity of the healthy foods has led to the revival of studies on phytochemicals activity. Plant substances, such as fiber regulate gastrointestinal tract function [1] and unsaturated fatty acids are capable of decreasing risk of atherosclerosis [2], and phenolic compounds can prevent oxidative stress [3]. All these compounds are crucial for the proper function of the human body. A number of important research papers concerning plant antioxidants were published in the last ten years, making an investigation of them an interesting branch of 21st century medical research [4,5,6].

Phenolic acids are a large group of secondary metabolites, originating from shikimic and benzoic acids [7]. They can be found commonly in plants, especially hydroxybenzoic and hydroxycinnamic derivatives, which are responsible for organoleptic properties, such as sour and bitter flavor. However, their true medicinal merit is an antioxidant and antiradical activity arising from their chemical structure. It is known that oxidative stress may cause damage to lipid membranes, DNA, and proteins [8], and may further lead to more severe diseases such as diabetes [9], Alzheimer’s. disease [10], and Parkinson’s disease [11] or neoplasms [12]. Each phenolic acid is made of one aromatic ring with hydroxyl residues and carboxyl residue linked to it. Acting as a donor of a hydrogen atom or single electron, they are capable of neutralizing reactive oxygen species (*ROS*), reducing transition metals responsible for Fenton’s reaction, and overall decreasing oxidative stress. Even though terms “antiradical” and “antioxidant” are often referred to the same property, these activities do not necessarily coincide. Thus, antiradical activity must be clearly distinguished from the antioxidant one [13]. While the first characterizes the ability of compounds to scavenge free radicals (for instance cation radical ABTS^•+^ and a stable radical DPPH^•^), the second one represents the ability to inhibit the process of oxidation. Measurement of antiradical activity most commonly is performed using ABTS or DPPH tests. During the ABTS method, proposed by Re et al. [14], pre-generated dark-green cation radical is reduced by a hydrogen-donating compound, such as phenolic acid. In the DPPH test [15], the radical undergoes a reaction with a reducer, becoming a neutral molecule. Both these assays rely on hydrogen exchange mechanism. As for the antioxidant activity, a wide variety of methods may be used, including biological assays, such as cellular antioxidant activity (CAA) [16] and chemical-based methods (FRAP [17], CUPRAC assays [18], etc.). While biological assays are considered to be more appropriate, they also are more expensive and time-consuming compare to chemical-based methods [16]. With all variety of methods results of efficiency measurements for phenolic acids is found to be slightly controversial in the literature [19,20]. 

Ferric Reducing Antioxidant Power assay (FRAP) [17] is based on reduction of a colorless Fe^3+^-TPTZ complex into intense blue Fe^2+^-TPTZ once it interacts with a potential antioxidant. At low cost, this method showed to be useful for screening of antioxidant capacities and comparing efficiencies of different compounds. Thus, in this study, we used FRAP method for an investigation of antioxidant activity of selected phenolic acids. Interestingly, the exact mechanism of the antioxidant activity for these compounds, and the influence of the compounds’ structure on their activity, is still not fully elucidated and controversial in the literature [21,22,23,24]. Computational chemistry proved to be a good support for experimental investigations. A great review of strategies in theoretical antioxidants activity research has been recently published by Galano et al. [25]. Not only hydrogen atom transfer (*HAT*) mechanism, but also sequential proton loss-electron transfer (*SPLET*) and single electron transfer-proton transfer (*SET-PT*) are widely studied with density functional theory (*DFT*) methods to elaborate the most probable mechanism of action of antioxidants [21,26,27]. Obtained results clearly showed that SPLET is the most favorable mechanism of action in a polar solvent, whilst HAT dominates in benzene. Presented computational results complement the experimental studies by explaining rationale used in the experiment. That indicates the importance of computational chemistry methods as a supporting tool in every modern-age research. 

Quantitative structure–activity relationship is widely used to find a relationship between structural features of compounds and their activities [28,29,30]. Finding proper descriptors used to develop a quantitative structure–activity relationship (QSAR) model is the very first step one should consider. For example, to build a model for wine polyphenols, Rastija et al. [31] used lipophilicity, Balaban index, Balaban-type index, and 3D GETAWAY descriptors. Gupta et al. [32] focused on MOLMAP descriptors selected by genetic algorithms. Filipović et al. [33] in their studies on free radical scavenging potency of 21 phenolic acids and simple phenolics proposed three models based on the number of vicinal hydroxyl groups, bond dissociation energy, proton affinity and electron transfer enthalpy. QSAR concept also was used by Chen et al. [21] in their studies on thermodynamic properties as descriptors for prediction of DPPH radical scavenging assay. These models provided a good insight into the nature of antiradical and antioxidant activity, though they require an involvement of quantum chemical calculations or other software for descriptor generation. The aim of this study is to perform a comprehensive investigation of antioxidants nature for the set of phenolic acids with different models of hydroxylation, and to develop a QSAR model for prediction of these properties based on a topology of tested compounds. FRAP assay has been used to measure antioxidant potential of selected phenolic acids. Purely topological descriptors used in this paper are easy to generate and give an understanding on how structural features of studied compounds influence an activity.

## 2. Results and Discussion

### 2.1. Experimental Results

Antioxidant properties of phenolic acids and their structures are presented in Table 1. The strongest activity reducing ion Fe^3+^ to Fe^2+^ was noted for 2,3-dihydroxybenzoic acid. The number of units per μmol of compound (*TAU_Fe/μmol_*) was equal to 202 ± 10.6. A slightly lower activity was observed for 3,4-dihydroxyphenylacetic (149 ± 10.0) and 2,5-dihydroxybenzoic acid (128 ± 6.3), while 3-hydroxybenzoic acid possessed the poorest efficiency among tested phenolic acids.

The statistical significance of differences between samples was analyzed using HSD Tukey test. Compounds have been divided into more active (1–11) and less active (12–22) set. Statistical significance was tested on level *p* < 0.05. Among the first group for nearly every pair except 8–9 and 10–11 (Table S1, Figure S1) the given criterion was achieved, whereas among the second group pairs 18–19, 18–20, 18–21, 18–22, 19–20, 19–21, 19–22, 20–21, 20–22, 21–22 didn’t met it (Table S2, Figure S2).

### 2.2. SAR Investigation

The results of measured antioxidant activities were used as a subject of structure–activity investigation. In order to explain the structure–activity relationship, three key factors must be investigated separately: mutual position of hydroxyl groups, their methylation, and the distance between phenyl and carboxylic group. These factors have been numerously mentioned in literature [21,22,34], however, the full clarification of their influence has not been reported.

### 2.3. Mutual Positions of Hydroxyl Groups and Resonance Stabilization

**Figure 1:**
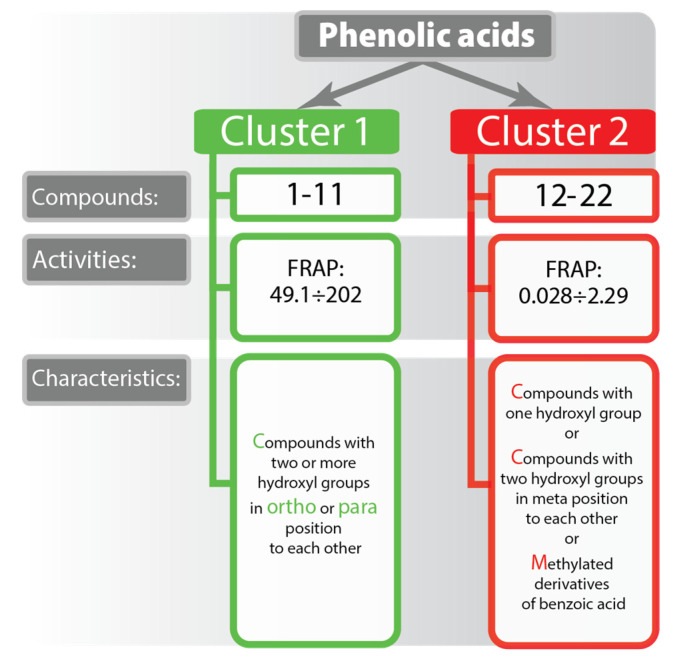
Phenolic acids clustered by their activity.

All tested phenolic acids can be clustered into two groups, one including compounds with high activities (**1**–**11**), and the other with compounds possessing extremely low activities (**12**–**22**) (Figure 1). Clustering of phenolic acids has shown a response dependence on the relative positions of the hydroxyl groups in the ring. Compounds, that contain only one hydroxyl group, (**13**, **15**–**18**, **20**, **22**) have exhibited very low efficiency. Phenolic acids with two hydroxyls substituted in the *meta* position in relation to each other, such as 3,5-dihydroxybenzoic (**19**) and 2,4-dihydroxybenzoic acid (**14**) have also shown a poor activity, compare to ones with two hydroxyls on adjacent carbon atoms.

Since FRAP assay must be performed in low pH values to maintain iron solubility, the ionization potential is also low, which drives hydrogen atom transfer. FRAP assay is based on an electron transfer mechanism with formation of aryloxyl radical [35]:

Fe(TPTZ)_2_^3+^ + ArOH → Fe(TPTZ)_2_^2+^ + ArO^•^ + H^+^

**Figure 2:**
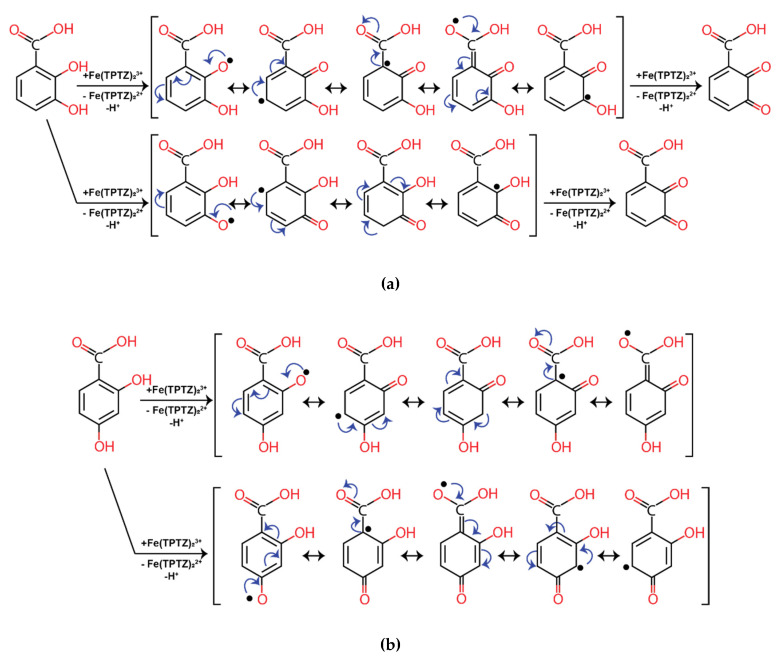
Resonance structures of phenolic acids: (**a**)—2,3-dihydroxybenzoic acid (**1**), and (**b**)—2,4-dihydroxybenzoic acid (**14**).

In this case, once aryloxyl radical is formed, its stability determines the efficiency of phenolic acid as an antioxidant compound. The fact that 2,4-dihydroxybenzoic acid (**14**) is much less efficient than 2,3-dihydroxybenzoic acid (**1**) can be explained by resonance structures of its radicals (Figure 2). In both cases radical is stabilized by delocalization over conjugated aromatic rings system. When the second hydroxyl group present in *ortho-* or *para-* position related to the first one, it allows second oxygen atom to participate in delocalization (Figure 2a). One can see that it leads to the activation of the second hydroxyl group. Hence, the reaction can proceed further reducing one more Fe^3+^-TPTZ complex with subsequent oxidation to 5,6-dioxo-1,3-cyclohexadiene-1-carboxylic acid. Highly active 2,5-dihydroxybenzoic acid (**3**) has the same resonance structures with hydroxyl groups being in *para*-position to each other. Meanwhile, the second hydroxyl of compound **14** (Figure 2b) does not participate in a delocalization, thus 2,4-dihydroxybenzoic acid exhibits significantly lower antioxidant activity. To summarize, in order to achieve high antioxidant and antiradical activity it is critical that two or more hydroxyl groups are located either in vicinal positions or on opposite sides of the ring (in *ortho* or *para* position to each other).

Low efficiency of two other compounds from the second cluster (4-hydroxy-3-methoxybenzoic acid (**12**) and 3,4-dimethoxybenzoic acid (**21**)) cannot be justified by mutual position of hydroxyl groups as similar in structure 3,4-dihydroxybenzoic acid (**10**) possesses relatively high efficiency as an antioxidant compound. Thus, methylation of hydroxyl groups must be considered separately. 

### 2.4. The Influence of Methylation

The results of FRAP tests showed a tendency of the methylated compounds to have lower activity than their non-methylated counterparts (**5** < **4**, **21** < **12** < **10**, **11** < **9**, **8** < **2**). Methylation yields a decrease of active electron- and hydrogen-donating groups, which consequently leads to reduced efficiency of the compound as an antioxidant. Interestingly, methylation of the cinnamic acid derivatives has no significant influence on the antioxidant activity, since 4-hydroxy-3-methoxycinnamic acid (**11**) demonstrates slightly lower activity than 3,4-dihydroxycinnamic one (**9**). With a decreasing distance between carboxylic group and a ring the influence of methylation is increasing, for example: 3,4-dihydroxyphenylacetic acid (**2**) is almost two times more efficient then its partially methylated counterpart (**8**), while the ratio between activities of 3,4-dihydroxybenzoic acid (**10**), 4-hydroxy-3-methoxybenzoic acid (**12**), and 3,4-dimethoxybenzoic one (**21**) amounts 598:26:1. It seems that the farther the carboxylic group is from the methoxylated ring, the more efficient the phenolic acid containing methylated hydroxyl groups. This can be explained by the importance of the inductive effect of carboxylic group. The 4-hydroxy-3,5-dimethoxybenzoic acid (**5**) is the only exception from the presented trend, it exhibits just slightly lower activity when compared to non-methylated counterparts (**4**). 

### 2.5. Carboxylic Group Influence and H-Bonding

Antioxidant activity decreases with an increase of the carboxylic group electron-withdrawing effect on a radical delocalization. In most cases, cinnamic acid derivatives have demonstrated improved efficiency over their counterparts derived from benzoic acid. Phenolic acids substituted with hydroxyls in *para-meta* position have their activity decreasing in the following order phenylacetic acid > cinnamic acid > benzoic acid (**2** > **9** > **10**, and **8** > **11** > **12**). This trend suggests that carboxylic group has the biggest influence on the total antioxidant activity. This influence occurs not through inductive effect (*-I*) (the distance of carboxylic group from the ring), but through the mesomeric effect (*-M*) (the resonance with the ring). Carboxylic group of phenylacetic acid is not participating in resonance and can influence only through -I effect, while carboxyl group of a benzoic acid shows the strongest -I and -M effects. Interestingly, phenolic acids that are substituted with only one hydroxyl in *para* position have demonstrated a decrease of activity presented in a row cinnamic acid > phenylacetic acid > benzoic acid (**15** > **17** > **20**), that may indicate the importance of inductive effect for low efficient compounds. Though, the difference in their efficiency is negligible. Meanwhile, an antioxidant activity of *ortho*-substituted compounds such as 2,5-dihydroxybenzoic acid (**3**) and 2-hydroxybenzoic acid (**13**) measured by FRAP have shown to be elevated when compared to its phenylacetic counterparts (2,5-dihydroxyphenylacetic (**7**) and 2-hydroxyphenylacetic (**16**) acids). In addition to the resonance stabilization (Figure 2), a radical can be stabilized by intermolecular hydrogen bonds between functional groups and polar protic solvents, as well as intramolecular hydrogen bonds [24]. There are two possible types of intramolecular hydrogen bonding for compounds tested here. One involves only hydroxyl oxygens, and the other is a hydrogen bond between carboxylic and hydroxyl groups.

One of the methods used for approximation of the hydrogen bonding energy is based on the calculation of potential energy density has been implemented in Multiwfn program package [36]. Energies (*E_HB_*) for all the studied compounds, where hydrogen bonding is possible, were calculated and collected in Table 2, along with distances between critical point (*CP*) and hydrogen atom (*H_HB_*), the distance between hydrogen (*H_HB_*) and acceptor oxygen atom (*O_ac_*) as well as the angle (*∠O_ac_-CP-H_HB_*).

The highest hydrogen bond energy has been found for bonds between hydrogen of hydroxyl group and double-bonded oxygen of benzoic acid’s carboxylic group (compound **1**, **3**, **13**, **14**). The lowest hydrogen bond energy is found to be between hydroxyl groups. According to the presented results, the oxygen of hydroxyl group is a less efficient H-bond acceptor when compared to double-bonded oxygen of carboxyl group. Taking into account this difference in H-bond strength, one can explain why for benzoic acid derivatives efficiency is decreasing in the row: *ortho-meta (2,3-pattern)* (**1**) > *ortho-meta (2,5-pattern)* (**3**) > *meta-meta-para* (**4**) > *meta-para* (**10**), while the same tendency does not work for phenylacetic and cinnamic acid derivatives. Similar to 2-hydroxybenzoic acid, 2,5-dihydroxyphenylacetic (**7**) also can be stabilized by a H-bond between carboxylic group and *ortho*-hydroxyl. However, surprisingly high H-bond energy (−11.41 kcal/mol) in the case of 2,5-dihydroxyphenylacetic one (**7**) does not justify its low efficiency compare to 3,4-dihydroxyphenylacetic acid (**2**), where energy of hydrogen bond between two hydroxyl groups is −5.47 kcal/mol. Moreover, due to the steric hindrance in case of the phenylacetic acid derivatives, H-bonding between carboxylic group and *ortho*-hydroxyl should not be that favorable as the one between two hydroxyl groups. 

**Figure 3:**
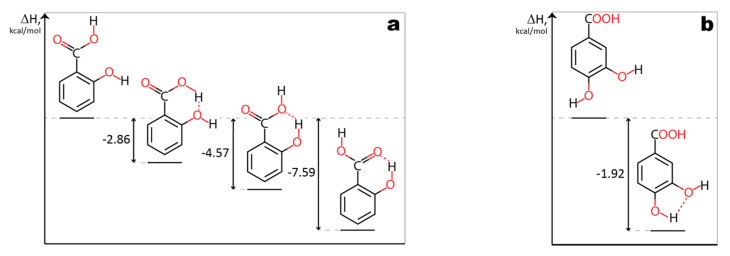

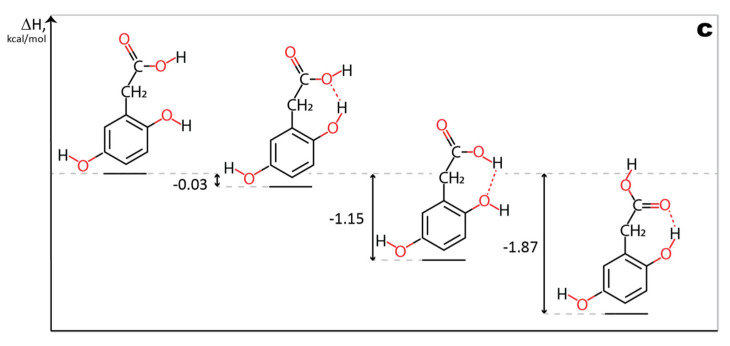
Enthalpies of hydrogen bond formation. (**a**)—2-hydroxybenzoic acid (**13**); (**b**)—3,4-dihydroxybenzoic acid (**10**); (**c**)—2,5-dihydroxyphenylacetic acid (**7**).

To verify results obtained by the method proposed in Multiwfn, an additional method was employed. Density functional theory was used to calculate enthalpies of H-bond formation. Formation of hydrogen bond in 2-hydroxybenzoic acid (**13**) results in release of 7.59 kcal/mol of energy (Figure 3a). The energy yield of H-bond formation in case of 3,4-dihydroxybenzoic acid (**10**) is significantly smaller (1.92 kcal/mol) (Figure 3b). These results are in good agreement with previously obtained data with Multiwfn method. Though, in the case of compound **7** (Figure 3c), H-bonding is not that energetically favorable (1.87 kcal/mol released) due to the steric tension occurring in this molecule. This finding is contrary to results calculated using Multiwfn method. 

**Figure 4:**
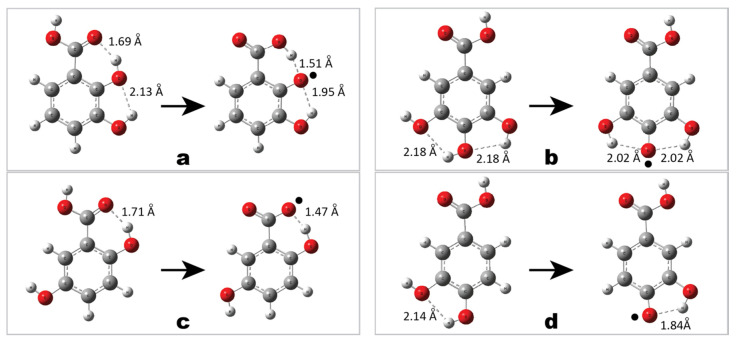
Intramolecular hydrogen bond stabilization of a molecule and its radical: (**a**)—2,3-dihydroxybenzoic acid (**1**); (**b**)—3,4,5-trihydroxybenzoic acid (**4**); (**c**)—2,5-dihydroxybenzoic acid (**3**); (**d**)—3,4-dihydroxybenzoic acid (**10**).

The 2,3-dihydroxybenzoic acid (**1**) (Figure 4a) may form two intramolecular hydrogen bonds: one involving only hydroxyl oxygen atoms and the other involving oxygen of carboxylic group (total of −18.47 kcal/mol, as is shown in Table 2). Similarly, two H-bonds stabilize a 3,4,5-trihydroxybenzoic acid (compound **4**, total of −7.30 kcal/mol) (Figure 4b). Though, in this case, the carboxylic group does not participate in H-bonding. The following compounds 2,5-dihydroxybenzoic acid (**3**) and 3,4-dihydroxybenzoic acid (**10**) (Figure 4c,d) are stabilized by just one intramolecular H-bond, and only in case of **3** strong interactions with oxygen of carboxylic group takes place (−12.45 kcal/mol, compared to −5.58 kcal/mol for compound **10**). It can be seen, that our study explained the trends in effectiveness. Hence, it is assumed that the model of phenolic acids’ hydroxylation by two hydroxyl groups, one of each is situated next to carboxyl group (in *ortho* position) may be efficient only for benzoic acid derivatives. 

### 2.6. QSAR Model

Original model, Multiple linear regression model, for the prediction of the antioxidant *TAU_Fe/μmol_* activity measured by FRAP assay had one outlier (4-hydroxy-3,5-dimethoxybenzoic acid). As it was earlier discussed this compound did not follow the general trend illustrated in SAR investigation. Thus, this point was excluded from the training set and model was rebuilt. Elimination of the 4-hydroxy-3,5-dimethoxybenzoic acid had no influence on selected descriptors, though it influenced their coefficients and statistical parameters. Finalized model based on four topological descriptors is represented by Equation (1):

(1) $$TAU_{Fe/\mu mol}=-0.507+62.03\cdot HOccOH\left(count\right)+74.83\cdot Ph_{ortho\left(para\right)-meta}+\;134.49\cdot B_{ortho-meta}+41.804\cdot C_{methyl}\left(count\right)$$

Descriptors used for MLR model have a clear chemical meaning and were in correspondence with the SAR investigation. First and foremost, the presence of HOccOH fragment is crucial. As it was indicated above, two or more hydroxyl groups in *ortho* position to each other positively impact an antioxidant activity due to charge delocalization and OH-OH intramolecular H-bonding. Not only information about mutual position of substituents, but also its methylation is decoded by this descriptor. Only non-methylated groups positively impact on efficiency. The evidence of phenylacetic acid derivatives being more efficient than others is supported by *Ph*_*ortho*(*para*)-*meta*_ descriptor, that indicates the presence of any *ortho-meta* or *para-meta* substitutes phenylacetic acids (compounds **2**, **7**, **8**). *B_ortho-meta_* is a specific descriptor that points out on a presence of *ortho*-*meta*-substituted benzoic acid derivatives (compounds **1** and **3**). The presence of strong intramolecular H-bonds between *ortho* hydroxyl and carboxylic groups results in significant increase of efficiency. Finally, *C_methyl_*(*count*) descriptor is correcting total activity calculated by the proposed model whilst taking into account the fact that methylation of cinnamic acid derivatives does not critically decrease an efficiency of these compounds. 

**Figure 5:**
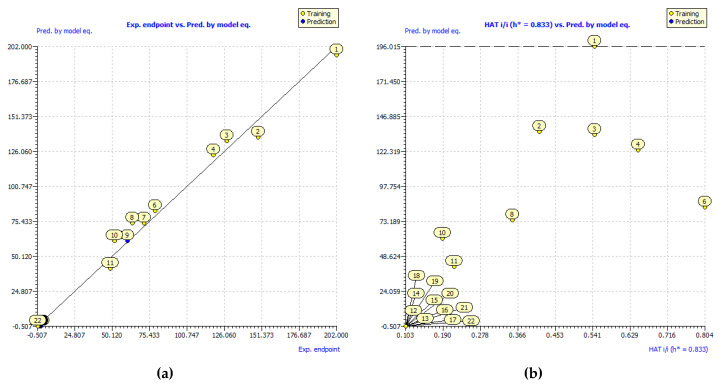
(**a**)—correlation plot between experimental and predicted values of $TAU_{Fe/\mu mol}$ antiradical activity; (**b**)—applicability domain of developed model. Yellow dots represent compounds selected as the training set, while the blue dots represent test set compounds.

Finalized model illustrates a good agreement with presented experimental results (Figure 5a) with correlation coefficient for the training set R = 0.9959. Interestingly, the mutual position of hydroxyl groups appeared to be an essential descriptor in several models for prediction of antioxidant activity proposed earlier. For instance, the simultaneous presence of the 3′,4′-dihydroxy structure at the B-ring or the adjustment of the hydroxyl group at the C-3 atom of selected flavonoid compounds was used among the other descriptors for prediction of inhibitory activity of Lipids peroxidation [37]. Using exclusively this descriptor Rasulev et al. developed a model with correlation coefficient R = 0.813. Meanwhile by adding Petijean shape index, dipole moment, and a number of Glycoside-like fragments as descriptors it was possible to increase a correlation coefficient to R = 0.938. The number of vicinal hydroxyl groups was used as a descriptor for MLR models in [33] developed for prediction of antiradical activity measured by ABTS assay and evaluated as vitamin C equivalent (VCEAC) for the set of 21 hydroxybenzoic acids and simple phenolic compounds. Models developed based on only this topological descriptor resulted in correlation coefficient R = 0.915. Meanwhile, by combining the number of vicinal hydroxyl groups with a bond dissociation enthalpy proton affinity, or with a proton affinity and an electron-transfer enthalpy as descriptors the accuracy of developed model was improved by R = 0.957 and R = 0.962, respectively. Model developed by us based purely on the topological descriptors achieved slightly better statistical parameters (Table 3) then earlier models. 

An applicability domain represents the response and chemical structure space in which the model makes predictions with a given reliability. All compounds from the second cluster (**12**–**22**) appeared to be on the border of a predicted applicability domain as well as compound **1** (Figure 5b). The location of these compounds close to an outlier region is determined by their extreme response values (extremely low for **12**–**22**, and extremely high for **1**). Nevertheless, all 21 phenolic acids were found inside an applicability domain. 

The number of statistical parameters that are used to validate developed models increase over years, as described in review by Gramatica et al. [38]. In this study, different validation criteria of robustness and predictivity have been used. The coefficient of determination R^2^ (the square of the sample correlation coefficient (R) between the experimental endpoint and predicted one), adjusted coefficient of determination R^2^_adj_ (in case when slope is set to zero), root mean square error *RMSE* (square root of the average of squared differences between predicted and experimental endpoint observation), mean absolute error *MAE* (the average magnitude of the errors in a set of predictions, without considering their direction) for both training and test sets were calculated. Additionally, specific coefficients of determination Q^2^_LOO_ and Q^2^_LMO_ (for leave-one-out and leave-many-out cross-validation, respectively) were calculated and presented in Table 3. Though, it must be noted, that even inadequate models with chance correlation may pass statistical analysis with sufficient values of parameters in case when it is built for data sets that include a small number of samples. When working with small data sets one must rely not only on pure statistics but mostly on a chemical knowledge about mechanism of activity of interest, and structure–activity relationship in order to select an adequate chemically justified model. 

With satisfactory statistical parameters and chemically justified descriptors (with *p*-values for all the descriptors equal to 0), developed here model, presumably, can well reflect the course of the reaction, and potentially be used for efficiency prediction of phenolic acids which activities are unknown. Based on both SAR and QSAR studies one can assume that 3,4,5-trihydroxyphenilacetic and 2,3,4-trihydroxybenzoic acids should be efficient as antioxidant compounds (Table 4), which is found to be in good agreement with the literature [39].

## 3. Materials and Methods 

### 3.1. Apparatus

Hitachi U 5100 spectrophotometer (Japan, Tokyo) connected with computer for controlling of measurement and data analyzing, temperature stabilizer, and glass cuvette with 1 cm optical path was used.

### 3.2. Reagents

#### 3.2.1. Phenolic Acids

Extrasynthese, Genay, France: 3,4-dihydroxyphenylacetic; 4-hydroxyphenylacetic; 4-hydroxy-3-methoxyphenylacetic; 2,5-dihydroxyphenylacetic; 4-hydroxycinnamic; 4-hydroxy-3-methoxycinnamic; 4-hydroxy-3,5-dimethoxycinnamic; 4-hydroxy-3-methoxybenzoic; 3,4,5-trihydroxybenzoic; 2-hydroxycinnamic; 3,4-dihydroxybenzoic.

Koch-Light Laboratories, Haverhill, United Kingdom: 3-hydroxycinnamic; 2,5-dihydroxybenzoic; 2,3-dihydroxybenzoic; 3,5-dihydroxybenzoic; 2,4-dihydroxybenzoic.

Fluka Chemie AG, Buchs, Switzerland: 3-hydroxybenzoic; 3,4-dimethoxybenzoic; 4-hydroxy-3,5-dimethoxybenzoic.

Fluka Chimica, Milano, Italy: 3,4-dihydroxycinnamic.

Sigma-Aldrich, St. Louis, MO, USA: 4-hydroxybenzoic; 2-hydroxybenzoic.

#### 3.2.2. Other Reagents

Merck, Darmstadt, Germany: methanol, gradient grade.

Chempur, Piekary Slaskie, Poland: methanol, pure for analysis; hydrochloric acid 35%, pure for analysis.

Sigma-Aldrich, St. Louis, MO, USA: sodium acetate trihydrate; iron (III) chloride; 2,2-diphenyl-1-picrylhydrazyl radical (*DPPH^•^*); 2,2′-azino-bis(3-ethylbenzothiazoline-6-sulfonic acid) diammonium salt (*ABTS*^•+^); potassium persulfate (*K_2_S_2_O_8_*).

Fluka, Buchs, Switzerland: 2,4,6-tris(2-pyridyl)-(S)-triazine (*TPTZ*).

### 3.3. Methods

#### 3.3.1. Measurement of Reducing Activity of Phenolic Acids with FRAP Method

The reducing activity of phenolic acids was measured with the Benzie and Strain method [17]. FRAP reagent was freshly prepared as follows: 12.5 mL of 0.3 mol/L acetate buffer (CH3COOH:CH3COONa), pH 3.6 was mixed with the same volume of methanol (Merck, Darmstadt, Germany). 2.5 mL of 10 mmol/L TPTZ in 0.04 mol/L HCl and 2.5 mL of 0.02 mol/L FeCl3·6H2O were added to such a solution.

225 μL of 50% solution of methanol in water and 75 μL of investigated phenolic compound solution (concentration chosen individually for each compound) were added to the 2.25 mL of FRAP reagent. The absorbance measurement at 593 nm was made at the beginning of the reaction and after 1 min at 37 °C. Measurements were repeated 5 times, for each phenolic acid. The results of experiments were presented as a reaction rate expressed as number of total antioxidant units per µmol of substance (*TAU_Fe/µmol_*). One unit is the amount of substance that reduces one µmol of Fe^3+^ to Fe^2+^ at 593 nm during 1 min at 37 °C. Measurements were done in a glass cuvette with 1 cm optical path. The number of units per 1 μmol of phenolic acid was calculated with the following Equation (2):

(2) $$TAU_{Fe/\mu mol}=\frac{1.513*A_{F0}-A_{F1}}{c}$$

where *TAU_Fe/μmol_*—is the number of units per µmol of phenolic acid; *A_F0_*—is the absorbance of the solution at the beginning of the reaction; *A_F1_*—is the absorbance of the solution after 1 min of the reaction; *c*—is the concentration of phenolic acid in the reaction mixture [*mM*].

The absolute error was calculated with the total differential method and the statistical significance of differences between samples was estimated using ANOVA with Tukey’s test, using Statistica version 13.3.

#### 3.3.2. DFT Calculations

Density functional theory was used to perform thermodynamic calculations in order to support hypothesis proposed in structure–activity relationship (*SAR*) investigations. Molecular structures of the studied compounds have been visualized using GaussView application [40] and optimized using B3LYP/cc-pVDZ level of theory with Gaussian16 [41] in an implicit water solvent, using conductor-like polarizable continuum model (*CPCM*) solvation method. Topology analysis of hydrogen-bonding was performed using Multiwfn 3.7 [36]. Hydrogen bond energy was calculated using Equation (3) [42]:

(3) $$E_{HB}=\frac{V\left(r_{bcp}\right)}{2}$$

where $V\left(r_{bcp}\right)$—is a potential energy density *V*(*r*) at corresponding bond critical point (*BCP*).

Additionally, relative intramolecular hydrogen bond enthalpy (*ΔH*) was calculated by comparing the sum of electronic and thermal enthalpies of the conformer with intramolecular hydrogen bonds and of the lowest-energy conformer without hydrogen bonds, following the procedure demonstrated by Korth et al. [43]. 

#### 3.3.3. Topological Descriptors

The choice of descriptors, which might be relevant to the activity of interest, is a problematic task. In this study, our aim was to build a QSAR model that is based exclusively on topology of phenolic acids. Optimized structures of studied compounds were saved in MDL MOL (*.mol*) format. PaDEL-Descriptor software [44] was used for the calculation of 2D topological descriptors and fingerprints. Calculated 2D descriptors included number of atoms and atoms of a certain element type, aromatic atoms, aromatic bonds, acidic and basic groups, hydrogen bond acceptors and donors, bonds of certain bond order, as well as topological descriptors characterizing the carbon connectivity, topological descriptors combining distance and adjacency information, etc. List of fingerprints calculated included Estate [45], Pubchem, Substructure [46], Klekota-Roth [47] and 2D atom pairs’ fingerprints. Additional specific sets of descriptors have been developed manually based on *ortho-para-meta-* substitution pattern of hydroxyl groups, effect of carboxyl group and methylation. A total of 12,355 descriptors were used.

#### 3.3.4. QSAR Model Development and Validation

QSARINS v2.2.4 [48,49] software (QSAR Research Unit in Environmental Chemistry and Ecotoxicology Department of Theoretical and Applied Sciences (DiSTA), University of Insubria, Varese, Italy) was used for data preparation and model development. The entire data set was preprocessed using a filter to eliminate constant (>80%) and codependent (>95%) descriptors. A splitting procedure plays a critical role for small data sets, since assigning an insufficient number of compounds into the validation set may result in the developed model being overtrained, while too many compounds in validation set may lead to loss of information for proper model development. Thus, only three compounds were selected randomly for validation set. Multiple linear regression (*MLR*) QSAR models were developed using the genetic algorithm (*GA*) method of variable subset selection. QSARINS software was also used for an applicability domain calculation by the leverage from the diagonal values of the Hat matrix. 

## 4. Conclusions

Antioxidant activity for a set of phenolic acids was measured by FRAP assay. SAR investigation showed that mono hydroxylated compounds and compounds with two hydroxyl groups in *meta* position to each other exhibited the lowest efficiency as antioxidants, while compounds with two or more hydroxyl groups in *ortho* or *para* position to each other illustrated the highest antioxidant properties. Methylated phenolic acids derivatives were shown to be less efficient compare to their nonmethylated counterparts. Two stabilization factors were elucidated: resonance stabilization of radical and intramolecular hydrogen bonding. Due to resonance stabilization, *ortho-meta* and *para-meta* hydroxylated phenolic acids have an improved activity over mono hydroxylated ones and those having two hydroxyl groups in *meta* position to each other. Hydrogen bonding stabilization explained the reason behind the elevated activity of benzoic acid derivatives with substituted *ortho* position. Proposed hypotheses have been validated by quantum chemical calculations.

Multiple Linear Regression model for the prediction of antioxidant activity measured by FRAP assay was built based on four topological descriptors that include the presence of HOccOH fragment, any *ortho-meta* or *para-meta* substitutes phenylacetic acids, *ortho*-*meta*-substituted benzoic acid derivatives, and the number of methylated fragments of cinnamic acid derivatives. All these descriptors were in correspondence with SAR investigation. Having just one outlier (4-hydroxy-3,5-dimethoxybenzoic acid), the developed model has satisfactory statistical parameters and can be used for activity prediction of new, not yet tested, phenolic acids based on their structural features. 

## Figures and Tables

**Table 1:** Structures of investigated phenolic acids.

	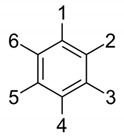							
**Compound ID:**	**IUPAC Name**	**C1**	**C2**	**C3**	**C4**	**C5**	**C6**	*TAU_Fe/μmol_* *
1	2,3-dihydroxybenzoic	COOH	OH	OH	H	H	H	202 ± 10.6
2	3,4-dihydroxyphenylacetic	CH_2_COOH	H	OH	OH	H	H	149 ± 10.0
3	2,5-dihydroxybenzoic	COOH	OH	H	H	OH	H	128 ± 6.3
4	3,4,5-trihydroxybenzoic	COOH	H	OH	OH	OH	H	119 ± 6.4
5	4-hydroxy-3,5-dimethoxybenzoic	COOH	H	OCH3	OH	OCH3	H	84.6 ± 3.7
6	4-hydroxy-3,5-dimethoxycinnamic	CH=CHCOOH	H	OCH3	OH	OCH3	H	79.2 ± 4.9
7	2,5-dihydroxyphenylacetic	CH_2_COOH	OH	H	H	OH	H	72.1 ± 3.3
8	4-hydroxy-3-methoxyphenylacetic	CH_2_COOH	H	OCH3	OH	H	H	63.9 ± 4.2
9	3,4-dihydroxycinnamic	CH=CHCOOH	H	OH	OH	H	H	60.9 ± 2.8
10	3,4-dihydroxybenzoic	COOH	H	OH	OH	H	H	52.0 ± 3.2
11	4-hydroxy-3-methoxycinnamic	CH=CHCOOH	H	OCH3	OH	H	H	49.1 ± 3.1
12	4-hydroxy-3-methoxybenzoic	COOH	H	OCH3	OH	H	H	2.29 ± 0.07
13	2-hydroxybenzoic	COOH	OH	H	H	H	H	2.01 ± 0.12
14	2,4-dihydroxybenzoic	COOH	OH	H	OH	H	H	1.30 ± 0.08
15	4-hydroxycinnamic	CH=CHCOOH	H	H	OH	H	H	0.777 ± 0.124
16	2-hydroxycinnamic	CH=CHCOOH	OH	H	H	H	H	0.556 ± 0.058
17	4-hydroxyphenylacetic	CH_2_COOH	H	H	OH	H	H	0.325 ± 0.081
18	3-hydroxycinnamic	CH=CHCOOH	H	OH	H	H	H	0.141 ± 0.044
19	3,5-dihydroxybenzoic	COOH	H	OH	H	OH	H	0.127 ± 0.044
20	4-hydroxybenzoic	COOH	H	H	OH	H	H	0.126 ± 0.030
21	3,4-dimethoxybenzoic	COOH	H	OCH_3_	OCH_3_	H	H	0.087 ± 0.049
22	3-hydroxybenzoic	COOH	H	OH	H	H	H	0.028 ± 0.032

* Averaged *TAU_Fe/μmol_* values with the maximal errors.

**Table 2:** Hydrogen bonding energies and geometrical parameters calculated using Multiwfn program package.

Compound ID:	IUPAC Name	E_HB_, (kcal/mol)	CP- H_HB_ Distance, (Å)	O_ac_ - H_HB_ Distance, (Å)	∠O_ac_-CP-H_HB_ Angle, (°)
1	2,3-dihydroxybenzoic	−12.91/−5.56	0.572/0.90	1.69/2.13	172.84/160.38
2	3,4-dihydroxyphenylacetic	−5.47	0.890	2.12	163.15
3	2,5-dihydroxybenzoic	−12.45	0.580	1.71	173.16
4	3,4,5-trihydroxybenzoic	−3.61/−3.69	0.882/0.880	2.18/2.18	161.43/162.80
5	4-hydroxy-3,5-dimethoxybenzoic	−6.32	0.836	2.06	167.15
6	4-hydroxy-3,5-dimethoxycinnamic	−6.37	0.833	2.06	167.30
7	2,5-dihydroxyphenylacetic	−11.41	0.601	1.78	176.03
8	4-hydroxy-3-methoxyphenylacetic	−6.16	0.842	2.07	167.46
9	3,4-dihydroxycinnamic	−5.42	0.902	2.13	161.51
10	3,4-dihydroxybenzoic	−5.58	0.882	2.11	163.67
11	4-hydroxy-3-methoxycinnamic	−6.30	0.835	2.06	167.47
12	4-hydroxy-3-methoxybenzoic	−6.26	0.838	2.06	167.31
13	2-hydroxybenzoic	−12.98	0.572	1.69	173.31
14	2,4-dihydroxybenzoic	−15.77	0.542	1.64	173.62

**Table 3:** Statistical parameters for developed model.

	R	R^2^	R^2^_adj_	RMSE	MAE	Q^2^_loo_	Q^2^_lmo_
training set	0.9959	0.9918	0.9893	5.5211	4.0678		
cross-validation				10.3056	7.4203	0.9716	0.9592
external validation	0.9996	0.9993	0.9973	1.5429	1.2627		

**Table 4:** Structures of phenolic acids and their antioxidant activities predicted by proposed quantitative structure–activity relationship (QSAR) model.

2D Structure of Tested Compounds	*TAU_Fe/μmol_* Predicted by MLR QSAR Model
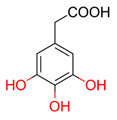	198.38
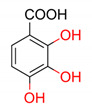	258.05

## Supplementary Materials

The Supporting Information is available online at Structures of investigated compounds in a .xyz format are accessible at the http://dx.doi.org/10.17632/77xhsdzk3y.

## References

[1] Anderson J.W., Baird P., Davis R.H., Ferreri S., Knudtson M., Koraym A., Waters V., Williams C.L. (2009). Health benefits of dietary fiber. Nutr. Rev..

[2] Lunn J., Theobald H.E. (2006). The health effects of dietary unsaturated fatty acids. Nutr. Bull..

[3] Zhang H., Tsao R. (2016). Dietary polyphenols, oxidative stress and antioxidant and anti-inflammatory effects. Curr. Opin. Food Sci..

[4] Pandey K.B., Rizvi S.I. (2009). Plant polyphenols as dietary antioxidants in human health and disease. Oxid. Med. Cell. Longev..

[5] Khan N., Afaq F., Mukhtar H. (2008). Cancer chemoprevention through dietary antioxidants: Progress and promise. Antioxid. Redox Signal..

[6] Kaliora A.C., Dedoussis G.V.Z., Schmidt H. (2006). Dietary antioxidants in preventing atherogenesis. Atherosclerosis.

[7] Riechmann J.L. (2002). Transcriptional Regulation: A Genomic Overview. Arab. B..

[8] Sies H., Berndt C., Jones D.P. (2017). Oxidative Stress. Annu. Rev. Biochem..

[9] Giacco F., Brownlee M. (2010). Oxidative stress and diabetic complications. Circ. Res..

[10] Wang X., Wang W., Li L., Perry G., Lee H., Zhu X. (2014). Oxidative stress and mitochondrial dysfunction in Alzheimer’s disease. Biochim. Biophys. Acta (BBA)-Molecular Basis Dis..

[11] Dias V., Junn E., Mouradian M.M. (2013). The role of oxidative stress in parkinson’s disease. J. Parkinsons. Dis..

[12] Reuter S., Gupta S.C., Chaturvedi M.M., Aggarwal B.B. (2010). Oxidative stress, inflammation, and cancer: How are they linked?. Free Radic. Biol. Med..

[13] Tirzitis G., Bartosz G. (2010). Determination of antiradical and antioxidant activity: Basic principles and new insights. Acta Biochim. Pol..

[14] Re R., Pellegrini N., Proteggente A., Pannala A., Yang M., Rice-Evans C. (1999). Antioxidant activity applying an improved ABTS radical cation decolorization assay. Free Radic. Biol. Med..

[15] Brand-Williams W., Cuvelier M.E., Berset C. (1995). Use of a free radical method to evaluate antioxidant activity. LWT Food Sci. Technol..

[16] López-Alarcón C., Denicola A. (2013). Evaluating the antioxidant capacity of natural products: A review on chemical and cellular-based assays. Anal. Chim. Acta.

[17] Benzie I.F.F., Strain J.J. (1996). The Ferric Reducing Ability of Plasma (FRAP) as a Measure of “Antioxidant Power”: The FRAP Assay. Anal. Biochem..

[18] Çelik S.E., Özyürek M., Güçlü K., Apak R. (2010). Solvent effects on the antioxidant capacity of lipophilic and hydrophilic antioxidants measured by CUPRAC, ABTS/persulphate and FRAP methods. Talanta.

[19] Rice-Evans C.A., Miller N.J. (1996). Antioxidant activities of flavonoids as bioactive components of food. Biochem. Soc. Trans..

[20] Yeh C.T., Yen G.C. (2003). Effects of phenolic acids on human phenolsulfotransferases in relation to their antioxidant activity. J. Agric. Food Chem..

[21] Chen Y., Xiao H., Zheng J., Liang G. (2015). Structure-thermodynamics-antioxidant activity relationships of selected natural phenolic acids and derivatives: An experimental and theoretical evaluation. PLoS ONE.

[22] Rice-Evans C.A., Miller N.J., Paganga G. (1996). Structure-antioxidant activity relationships of flavonoids and phenolic acids. Free Radic. Biol. Med..

[23] Lucarini M., Pedrielli P., Pedulli G.F., Cabiddu S., Fattuoni C. (1996). Bond dissociation energies of O-H bonds in substituted phenols from equilibration studies. J. Org. Chem..

[24] Amorati R., Valgimigli L. (2012). Modulation of the antioxidant activity of phenols by non-covalent interactions. Org. Biomol. Chem..

[25] Galano A., Raúl Alvarez-Idaboy J. (2019). Computational strategies for predicting free radical scavengers’ protection against oxidative stress: Where are we and what might follow?. Int. J. Quantum Chem..

[26] Milenković D., Đorović J., Jeremić S., Dimitrić Marković J.M., Avdović E.H., Marković Z. (2017). Free radical scavenging potency of dihydroxybenzoic acids. J. Chem..

[27] Markovic Z., DJOROVIC J., MARKOVIC J.M.D., Biocanin R., Amic D. (2016). Comparative density functional study of antioxidative activity of the hydroxybenzoic acids and their anions. Turkish J. Chem..

[28] Kubinyi H. (1997). QSAR and 3D QSAR in drug design. Part 1: Methodology. Drug Discov. Today.

[29] Puzyn T., Rasulev B., Gajewicz A., Hu X., Dasari T.P., Michalkova A., Hwang H.-M., Toropov A., Leszczynska D., Leszczynski J. (2011). Using nano-QSAR to predict the cytotoxicity of metal oxide nanoparticles. Nat. Nanotechnol..

[30] Hansch C., Hoekman D., Leo A., Zhang L., Li P. (1995). The expanding role of quantitative structure-activity relationships (QSAR) in toxicology. Toxicol. Lett..

[31] Rastija V., Medić-Šarić M. (2009). QSAR study of antioxidant activity of wine polyphenols. Eur. J. Med. Chem..

[32] Gupta S., Matthew S., Abreu P.M., Aires-De-Sousa J. (2006). QSAR analysis of phenolic antioxidants using MOLMAP descriptors of local properties. Bioorganic Med. Chem..

[33] Filipović M., Marković Z., Đorović J., Marković J.D., Lučić B., Amić D. (2015). QSAR of the free radical scavenging potency of selected hydroxybenzoic acids and simple phenolics. Comptes Rendus Chim..

[34] Cai Y.Z., Sun M., Xing J., Luo Q., Corke H. (2006). Structure-radical scavenging activity relationships of phenolic compounds from traditional Chinese medicinal plants. Life Sci..

[35] Gupta D. (2015). Methods for determination of antioxidant capacity: A review. Int. J. Pharm. Sci. Res..

[36] Lu T., Chen F. (2012). Multiwfn: A multifunctional wavefunction analyzer. J. Comput. Chem..

[37] Rasulev B.F., Abdullaev N.D., Syrov V.N., Leszczynski J. (2005). A Quantitative Structure-Activity Relationship (QSAR) Study of the Antioxidant Activity of Flavonoids. QSAR Comb. Sci..

[38] Gramatica P., Sangion A. (2016). A historical excursus on the statistical validation parameters for QSAR models: A clarification concerning metrics and terminology. J. Chem. Inf. Model..

[39] Siquet C., Paiva-Martins F., Lima J.L.F.C., Reis S., Borges F. (2006). Antioxidant profile of dihydroxy- and trihydroxyphenolic acids—A structure-activity relationship study. Free Radic. Res..

[40] Dennington R., Keith T.A., Millam J.M. (2016). GaussView 2016.

[41] Frisch M.J., Trucks G.W., Schlegel H.B., Scuseria G.E., Robb M.A., Cheeseman J.R., Scalmani G., Barone V., Petersson G.A., Nakatsuji H. Gaussian 16 2016. https://gaussian.com/gaussian16/.

[42] Espinosa E., Molins E., Lecomte C. (1998). Hydrogen bond strengths revealed by topological analyses of experimentally observed electron densities. Chem. Phys. Lett..

[43] Korth H.-G., de Heer M.I., Mulder P. (2002). A DFT study on intramolecular hydrogen bonding in 2-substituted phenols: Conformations, enthalpies, and correlation with solute parameters. J. Phys. Chem. A.

[44] Yap C.W. (2011). PaDEL-descriptor: An open source software to calculate molecular descriptors and fingerprints. J Comput. Chem..

[45] Hall L.H., Kier L.B. (1995). Electrotopological state indices for atom types: A novel combination of electronic, topological, and valence state information. J. Chem. Inf. Comput. Sci..

[46] Laggner C. (2009). SMARTS Patterns for Functional Group Classification. http://code.google.com/p/semanticchemistry/source/browse/wiki/InteLigand.wiki?spec=svn41&r=41.

[47] Klekota J., Roth F.P. (2008). Chemical substructures that enrich for biological activity. Bioinformatics.

[48] Gramatica P., Chirico N., Papa E., Cassani S., Kovarich S. (2013). QSARINS: A new software for the development, analysis, and validation of QSAR MLR models. J. Comput. Chem..

[49] Gramatica P., Cassani S., Chirico N. (2014). QSARINS-chem: Insubria datasets and new QSAR/QSPR models for environmental pollutants in QSARINS. J. Comput. Chem..

