# Supplementary material for: Antioxidant Activity of Selected Phenolic Acids–Ferric Reducing Antioxidant Power Assay and QSAR Analysis of the Structural Features
Source: Molecules. 2020 Jul 7;25(13):3088. doi: 10.3390/molecules25133088 (PMC7412039; doi:10.3390/molecules25133088)
Supplement: Supplementary file 1 [file molecules-25-03088-s001.pdf]

molecules

Supplementary Materials

Antioxidant Activity of Selected Phenolic Acids – Ferric Reducing Antioxidant Power Assay and QSAR Analysis of the Structural Features

Maciej Spiegel [1], Karina Kapusta [2,*], Wojciech Kołodziejczyk [2], Julia Saloni [2], Beata Żbikowska [1], Glake A. Hill [2] and Zbigniew Sroka [1]

[1] Department of Pharmacognosy, Wroclaw Medical University, Borowska 211, 50-556 Wroclaw, Poland
[2] Interdisciplinary Center for Nanotoxicity, Department of Chemistry, Physics and Atmospheric Sciences, Jackson State University, 1400 J. R. Lynch str., Jackson, MS, USA, 39217
* Correspondence: karina.kapusta@icnanotox.org (K.K.)

Table S1. HSD Tukey test results for the more active compounds (1-11).

#	1	2	3	4	5	6	7	8	9	10	11
**1**		0.000178	0.000178	0.000178	0.000178	0.000178	0.000178	0.000178	0.000178	0.000178	0.000178
**2**	0.000178		0.000178	0.000178	0.000178	0.000178	0.000178	0.000178	0.000178	0.000178	0.000178
**3**	0.000178	0.000178		0.000178	0.000178	0.000178	0.000178	0.000178	0.000178	0.000178	0.000178
**4**	0.000178	0.000178	0.000178		0.000178	0.000178	0.000178	0.000178	0.000178	0.000178	0.000178
**5**	0.000178	0.000178	0.000178	0.000178		0.005879	0.000178	0.000178	0.000178	0.000178	0.000178
**6**	0.000178	0.000178	0.000178	0.000178	0.005879		0.000246	0.000178	0.000178	0.000178	0.000178
**7**	0.000178	0.000178	0.000178	0.000178	0.000178	0.000246		0.000181	0.000178	0.000178	0.000178
**8**	0.000178	0.000178	0.000178	0.000178	0.000178	0.000178	0.000181		0.437086	0.000178	0.000178
**9**	0.000178	0.000178	0.000178	0.000178	0.000178	0.000178	0.000178	0.437086		0.000178	0.000178
**10**	0.000178	0.000178	0.000178	0.000178	0.000178	0.000178	0.000178	0.000178	0.000178		0.488280
**11**	0.000178	0.000178	0.000178	0.000178	0.000178	0.000178	0.000178	0.000178	0.000178	0.488280	

Table S2. HSD Tukey test results for the less active compounds (12-22).

#	12	13	14	15	16	17	18	19	20	21	22
**12**		0,000181	0,000178	0,000178	0,000178	0,000178	0,000178	0,000178	0,000178	0,000178	0,000178
**13**	0,000181		0,000178	0,000178	0,000178	0,000178	0,000178	0,000178	0,000178	0,000178	0,000178
**14**	0,000178	0,000178		0,000178	0,000178	0,000178	0,000178	0,000178	0,000178	0,000178	0,000178
**15**	0,000178	0,000178	0,000178		0,000607	0,000178	0,000178	0,000178	0,000178	0,000178	0,000178
**16**	0,000178	0,000178	0,000178	0,000607		0,000357	0,000178	0,000178	0,000178	0,000178	0,000178
**17**	0,000178	0,000178	0,000178	0,000178	0,000357		0,006340	0,002434	0,002359	0,000286	0,000179
**18**	0,000178	0,000178	0,000178	0,000178	0,000178	0,006340		1,000000	1,000000	0,974122	0,300822
**19**	0,000178	0,000178	0,000178	0,000178	0,000178	0,002434	1,000000		1,000000	0,997610	0,498697
**20**	0,000178	0,000178	0,000178	0,000178	0,000178	0,002359	1,000000	1,000000		0,997836	0,505833
**21**	0,000178	0,000178	0,000178	0,000178	0,000178	0,000286	0,974122	0,997610	0,997836		0,960114
**22**	0,000178	0,000178	0,000178	0,000178	0,000178	0,000179	0,300822	0,498697	0,505833	0,960114	

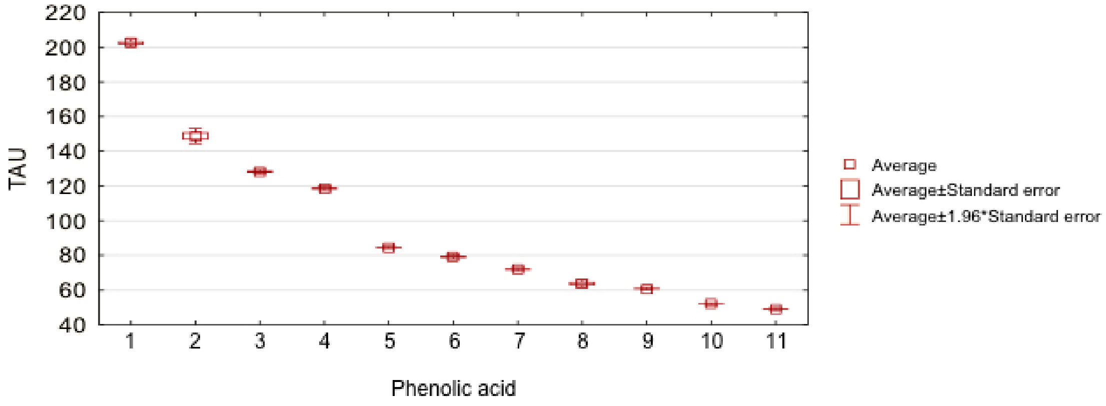

Figure S1. Visualization of HSD Tukey test results for the more active compounds (1-11).

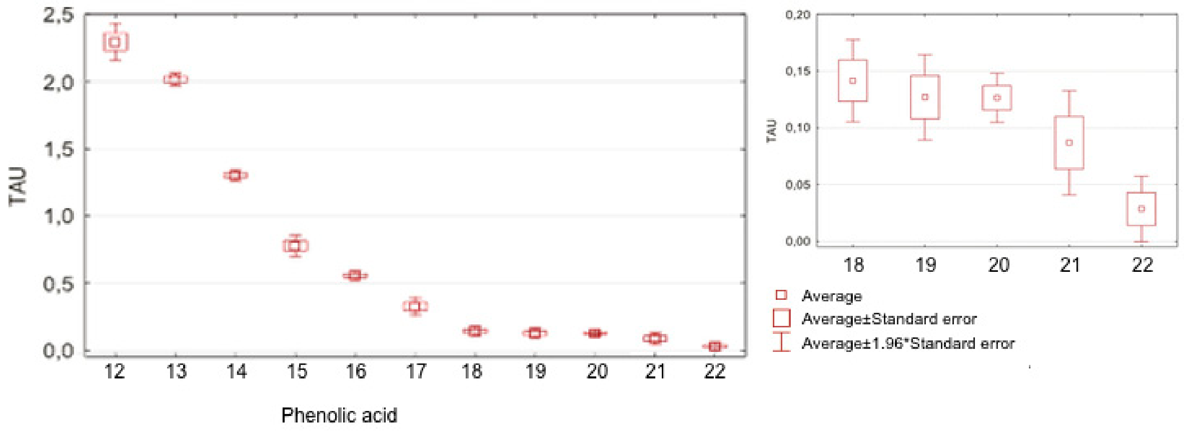

Figure S2. Visualization of HSD Tukey test results for the less active compounds (12-22).

© 2020 by the authors. Submitted for possible open access publication under the terms and conditions of the Creative Commons Attribution (CC BY) license (http://creativecommons.org/licenses/by/4.0/).
